# Supplementary material for: PRC2 promotes canalisation during endodermal differentiation
Source: PLoS Genet. 2025 Jan 30;21(1):e1011584. doi: 10.1371/journal.pgen.1011584 (PMC11813121; doi:10.1371/journal.pgen.1011584)
Supplement: S1 Fig — A) Targeting strategies employed for the construction of the B6 GSC-GFP and HHEX-RedStar double reporter mESC line [31,35]. B) Fluorescent and brightfield images (upper panel) and FACS scatter plot profiles (lower panel) of B6 reporter ESCs undergoing ADE differentiation on days 3–6. FACS profiles include GSC-GFP and HHEX-RedStar reporter fluorescence and immunofluorescent detection of the definitive mesendoderm marker CXCR4 (using anti-CXCR4-APC). Scale bars; 100 μm. C) FACs analysis profiles to determine sample drift between the indicated gates following the FACS collection period (≤ 2 h). Percentages in the original gates and in the post sort analysis are indicated. D) Quantitative RT PCR analysis of Gsc, Hhex, Pou5f1 and Cer1. Relative expression between differentiation samples was calculated by normalizing the transcript number by the geometric mean of Tbp, Pgk1 and Sdha expression. Error bars represent the standard deviation of the mean of ≥ 3 independent experiments. (PDF) [file pgen.1011584.s001.pdf]

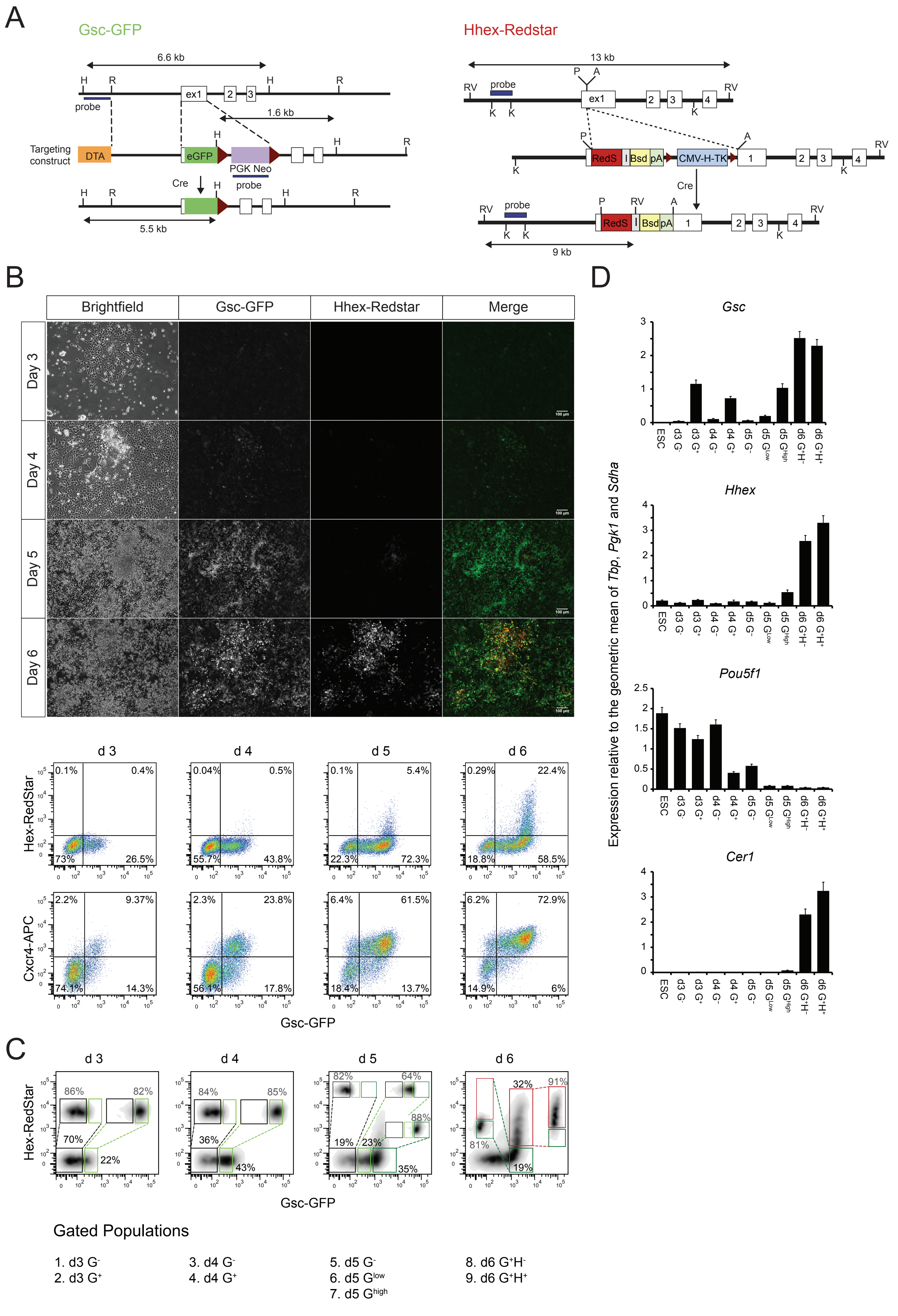
A
Gsc-GFP
Hhex-Redstar
6.6 kb
probe
ex1
1.6 kb
Targeting construct
DTA
eGFP
PGK Neo
Cre
5.5 kb
13 kb
RedS
Bsd
pA
CMV-H-TK
9 kb
B
Brightfield
Gsc-GFP
Hhex-Redstar
Merge
Day 3
Day 4
Day 5
Day 6
d 3
d 4
d 5
d 6
Hex-RedStar
Cxcr4-APC
Gsc-GFP
C
Gated Populations
1. d3 G-
2. d3 G+
3. d4 G-
4. d4 G+
5. d5 G-
6. d5 Glow
7. d5 Ghigh
8. d6 G+H-
9. d6 G+H+
D
Gsc
Hhex
Pou5f1
Cer1
Expression relative to the geometric mean of Tbp, Pgk1 and Sdha
