# Supplementary figures and images for: PRC2 promotes canalisation during endodermal differentiation
Source: PLoS Genet. 2025 Jan 30;21(1):e1011584. doi: 10.1371/journal.pgen.1011584 (PMC11813121; doi:10.1371/journal.pgen.1011584)

**A**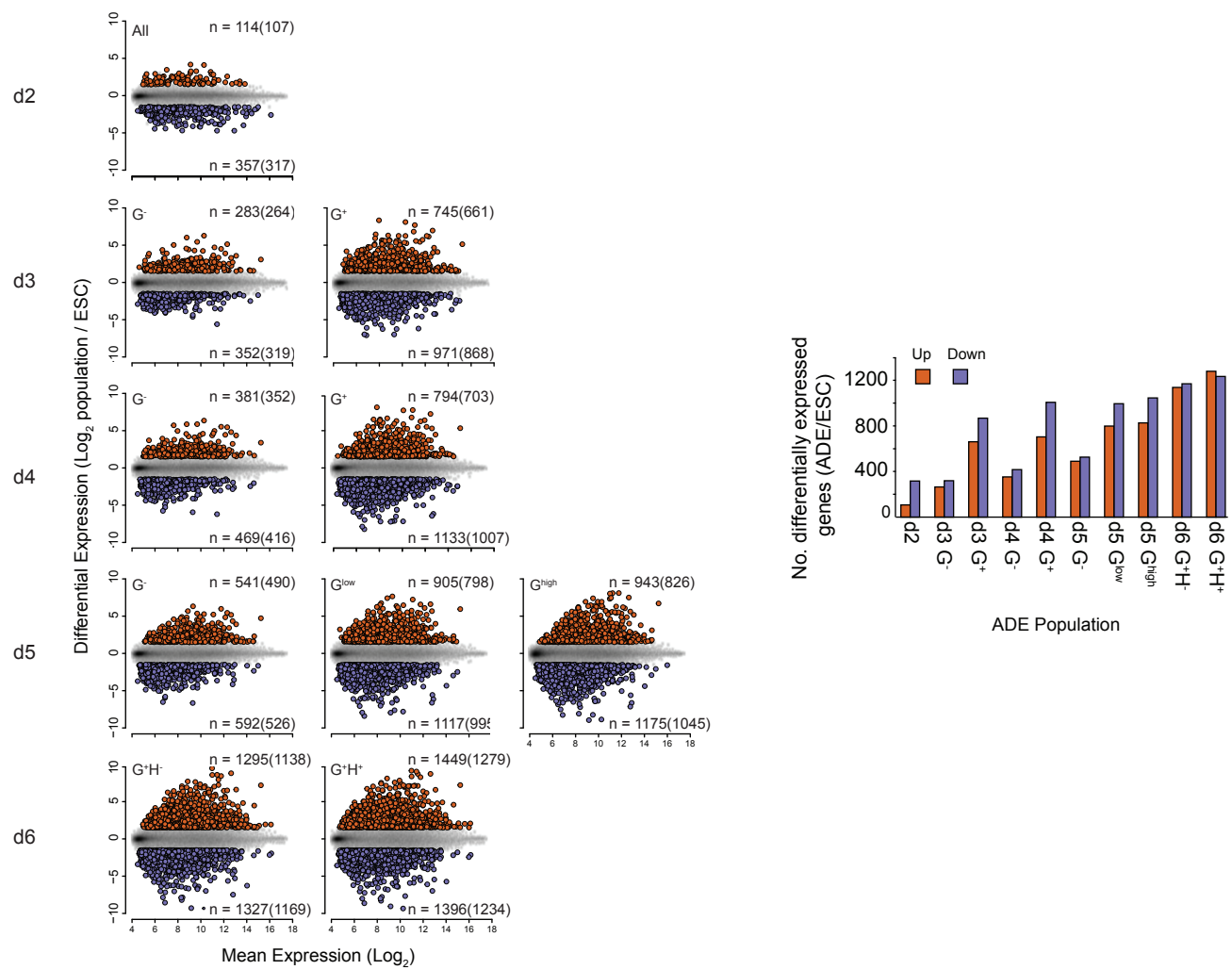**B**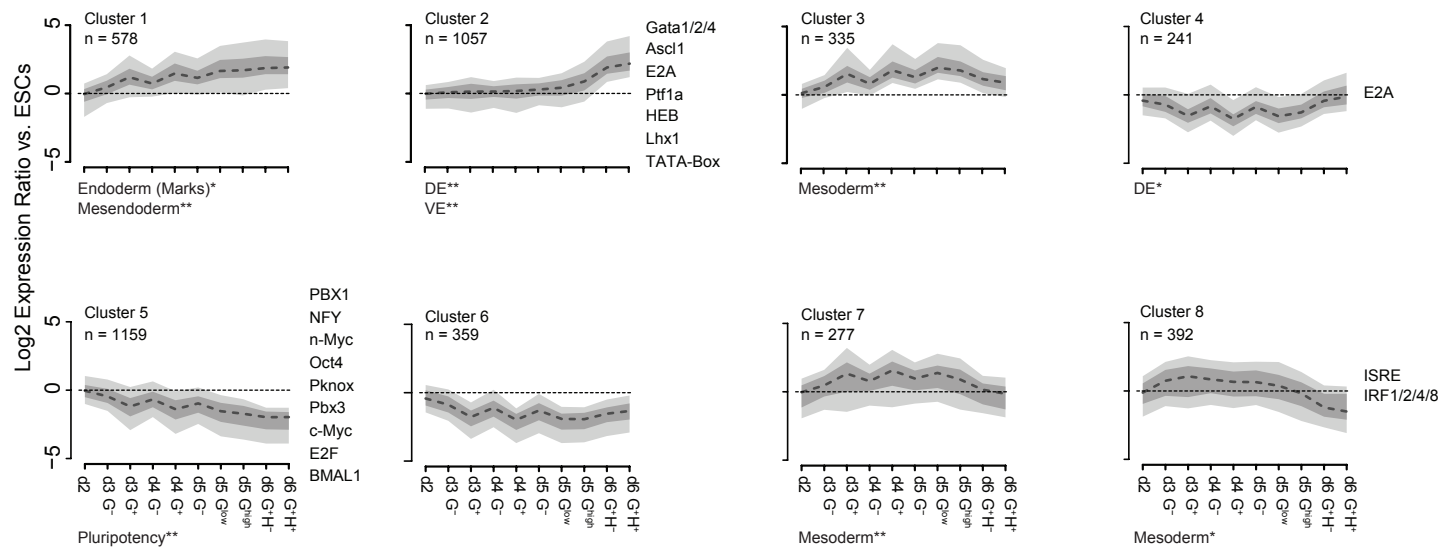

Supplement: S2 Fig — A) Pairwise scatter plots (left panel) and summary barplot (right panel) showing the number of differentially expressed genes between each ADE population vs. ESCs. Upregulated and down regulated genes are shown in red and blue respectively. The number of significant genes are noted in parenthesis (RefSeq annotation). Differentially expressed genes are defined as those with a log2 fold change of ≥ 1.5 and an adjusted p value of ≤ 0.01 (Benjamini & Hochberg multiple testing correction). B) Plots depicting the aggregate expression profile of the genes in each of the clusters defined in Fig 1E. The heavy dashed line, dark grey shaded area and light grey shaded areas represent the median, 25th to 75th percentile range and 10th to 90th percentile range of the log2 fold change for each gene set respectively. Significantly enriched functional gene sets for each of the clusters are indicated below their respective plot (p≤0.05* and p≤0.01** using a Fischer’s exact test with Benjamini & Hochberg multiple testing correction). Significantly enriched transcription factor motifs proximal to the TSSs (+100 bp to -50bp) are indicated to the right of each plot (p≤ 0.01 Benjamini multiple testing correction). (PDF) [file pgen.1011584.s002.pdf]

A

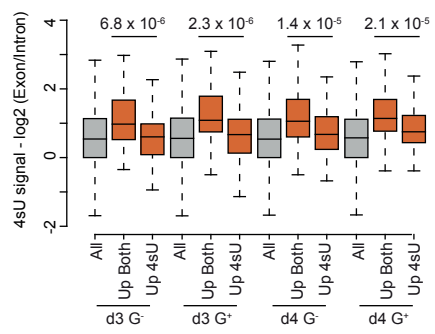

Supplement: S3 Fig — A) Boxplots showing length corrected log2 exon/intron ratios for all (grey) or upregulated (red; in d3 G+ vs. G-) for the indicated populations. Significant changes in log2 ratios were determined using a Wilcoxon rank-sum test (p values as displayed). Data represents the mean of three independent replicate experiments. (PDF) [file pgen.1011584.s003.pdf]

A

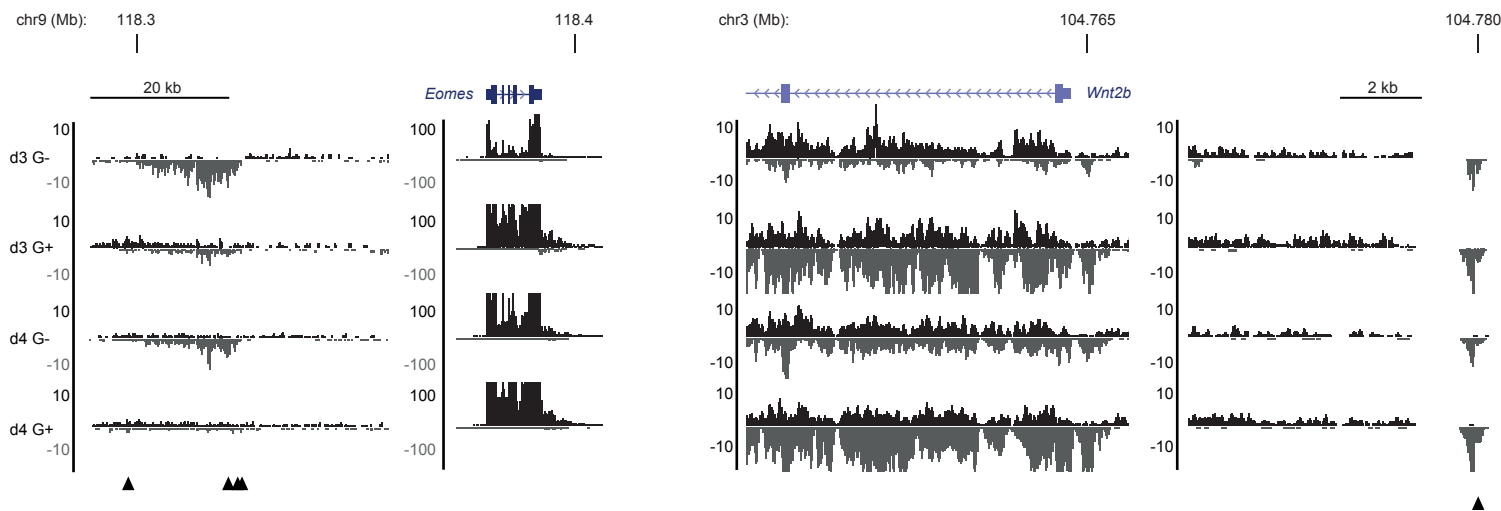

B

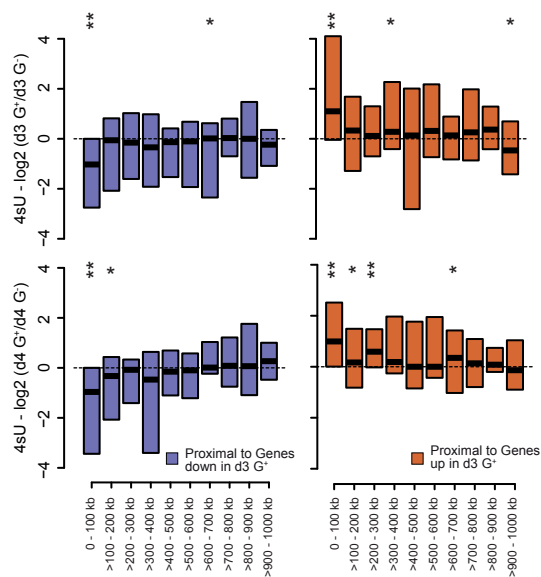

Supplement: S4 Fig — A) Genome browser tracks of normalised 4sU-seq signal at candidate gene loci with differentially expressed dREG peaks proximal to differentially regulated genes. Data and genes are presented as in Fig 2A and dREG peaks are indicated with black arrow heads. B) Boxplots showing the log2 ratio (G+/G-) of normalised 4sU-seq signal for dREG peaks across a range of distance separations from differentially regulated genes (in d3 G+ vs. G-). Plots show the log2 ratios for d3 and d4 of differentiation (upper and lower panels respectively) at dREG peaks associated with downregulated (blue) and upregulated genes (red). Boxplots depict the median and 25%-75% data distribution (black bar and coloured box respectively). Significant changes between the populations for each distance and condition for the raw, unlogged values, are indicated above their respective plot (p≤0.05* and p≤0.01** determined using a Wilcoxon signed rank test). Data shown in (A) and (B) represent the mean of three independent replicate experiments. (PDF) [file pgen.1011584.s004.pdf]

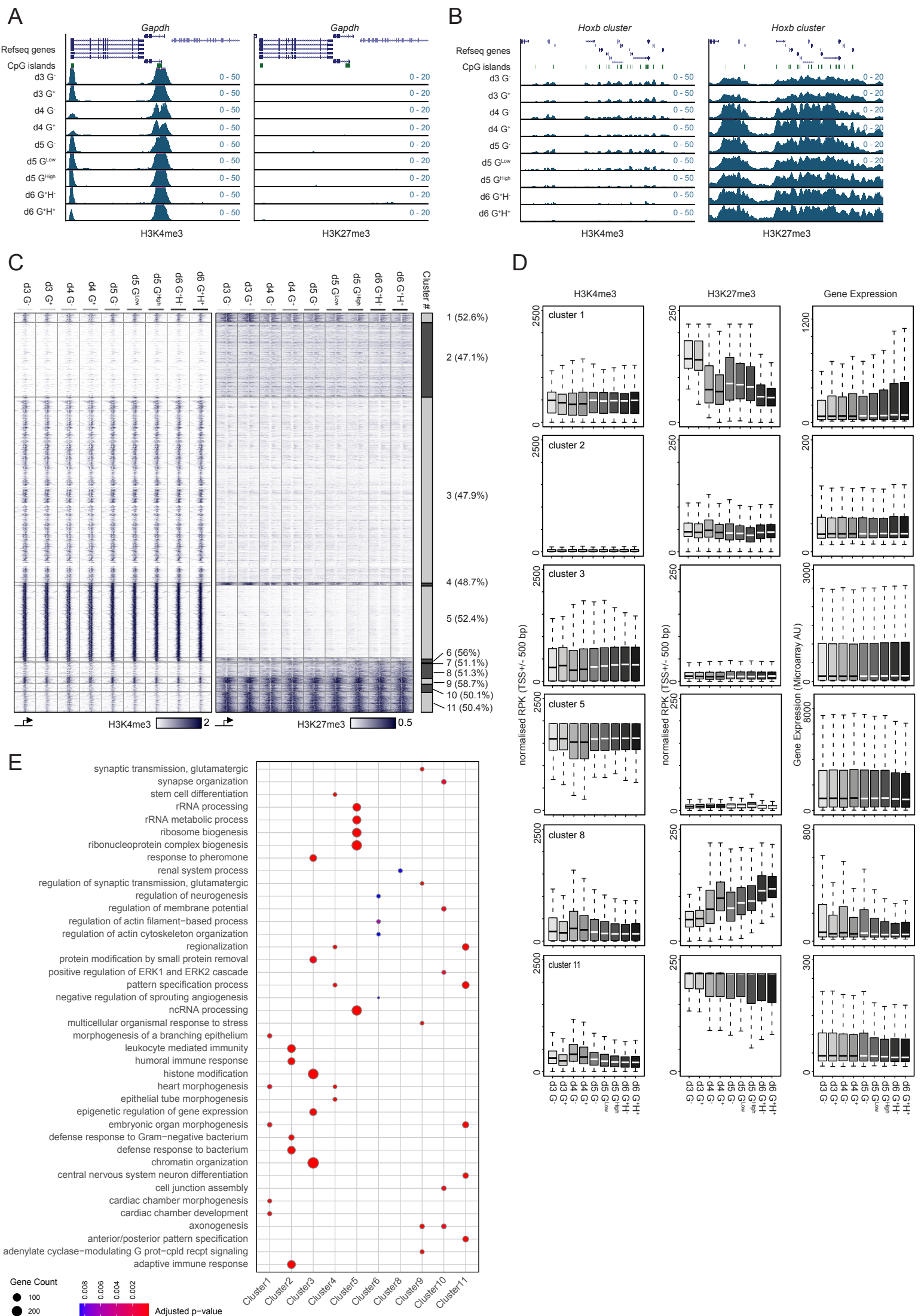

Supplement: S5 Fig — Genome browser tracks of ChIP-seq signal for H3K4me3 (left panel) and H3K27me3 (right panel) at the A) Gapdh and B) Hoxb gene loci (annotated as per the mm9 genome assembly). C) Heatmaps of H3K4me3 (left) and H3K27me3 (right) ChIP-seq signal spanning +/- 5 kb of gene TSSs grouped and ordered based on clustering of the TSS (+/- 500 bp) ChIP-seq signal from d3 G- and d6 G+H+ populations for both histone modifications. Heatmaps are sub-divided into 11 clustered groups, and the percentage of genes with a CGI TSS in each group is shown in parenthesis. The signal scale for each modification is shown below their respective heatmap. D) Summary boxplots of normalised H3K4me3 and H3K27me3 ChIP-seq signal at the TSSs (+/- 500 bp; left and middle panel respectively) and gene expression levels (right panel) for six representative clusters from panel (D). E) The top five enriched functional gene ontology terms (biological process) for each of the ChIP-defined clusters shown in (C; N.B. cluster seven is absent as it lacked any significantly enriched terms). Gene count and adjusted p-values are indicated as per the key. Full lists of enriched functional terms for each cluster are provided in S6 Table. ChIP-seq data shown in (A—D) and gene expression data shown in (D) represent the mean of two and three independent replicate experiments respectively. (PDF) [file pgen.1011584.s005.pdf]

**A**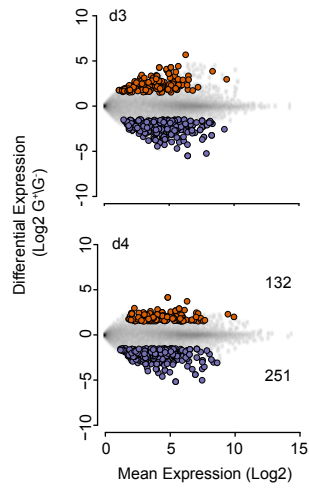**C**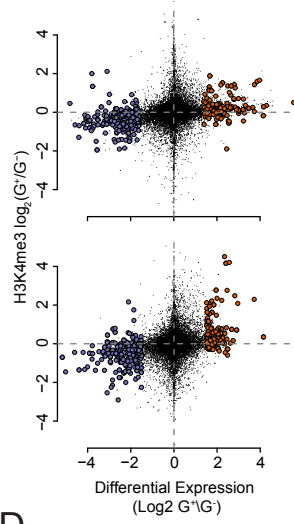**E**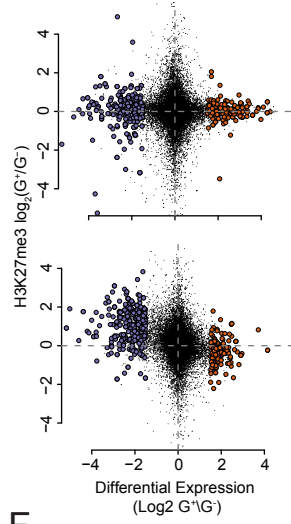**B**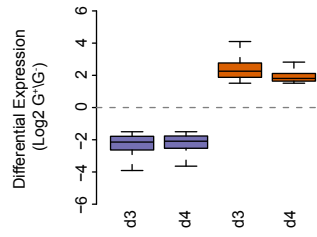**D**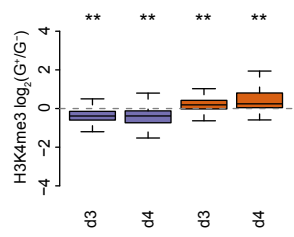**F**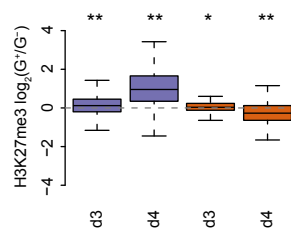

Supplement: S6 Fig — A) MA plots of gene sets that show consistent differential transcription/4sU-seq signal between the G+ and G- populations at d3 and d4 of ADE differentiation. Upregulated and downregulated genes are coloured in red and blue respectively and the number of genes are noted in parenthesis in the lower panel. B) Summary boxplots of log2 G+/G- ratios for the gene and datasets shown in panel A. C) Scatter plots depicting the log2 G+/G- transcription/4sU-seq signal ratios (x axis) vs. the log2 G+/G- ratio of H3K4me3 ChIP-seq signal at the TSS (+/- 500bp; y axis) of the differentially transcribed genes shown in A. D) Summary boxplots of the log2 G+/G- ratio of H3K4me3 ChIP-seq signal at the TSS (+/- 500 bp; y axis) for the gene and datasets shown in (C). Significant changes between the populations for each day for the raw, unlogged values, are indicated above their respective plot (p≤0.05* and p≤0.01** determined using a Wilcoxon signed rank test). E) As for (C) but for H3K27me3 ChIP-seq data. F) As for (D) but for H3K27me3 ChIP-seq data. 4sU-seq data (A and B) and ChIP-seq data (C—F) represent the mean of three and two independent replicate experiments respectively. (PDF) [file pgen.1011584.s006.pdf]

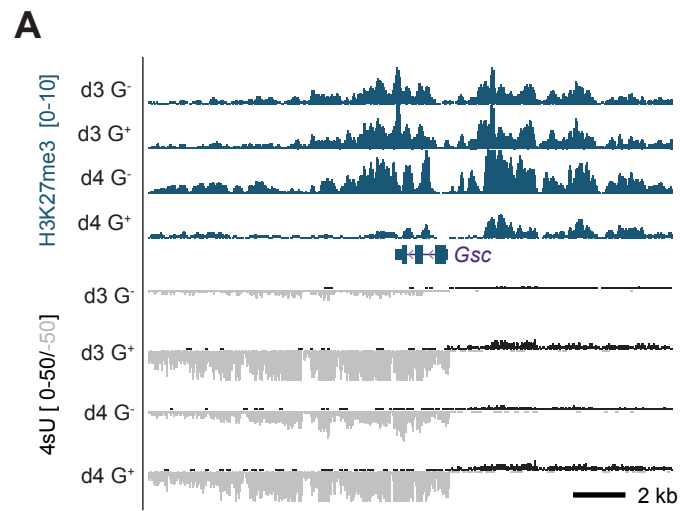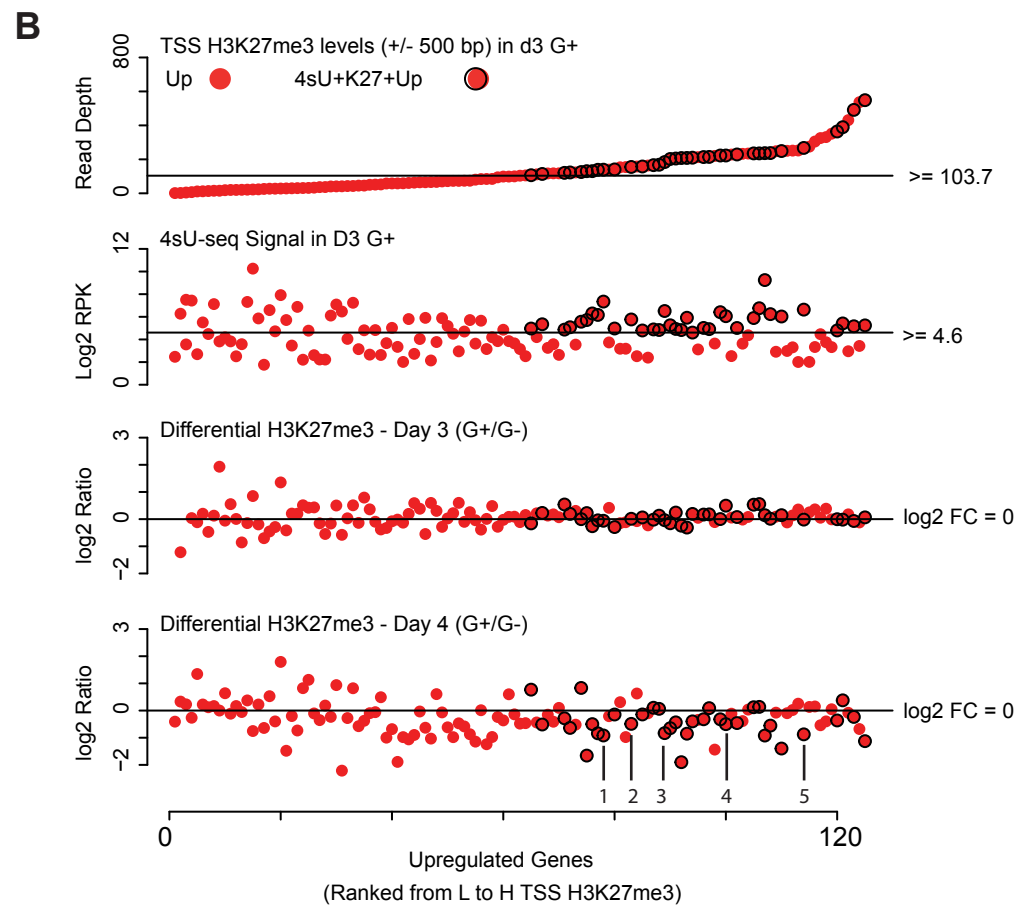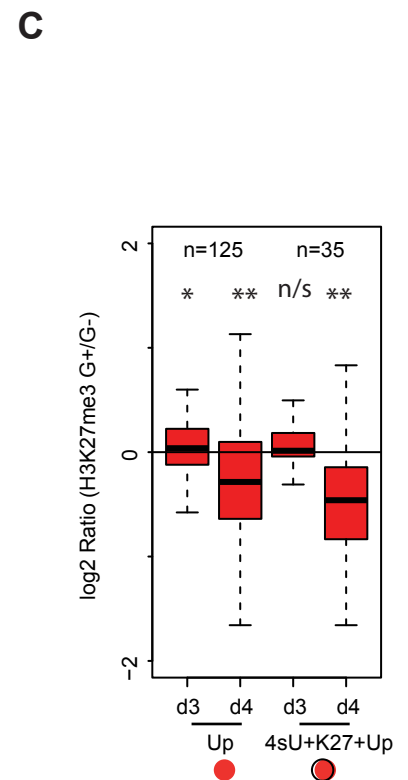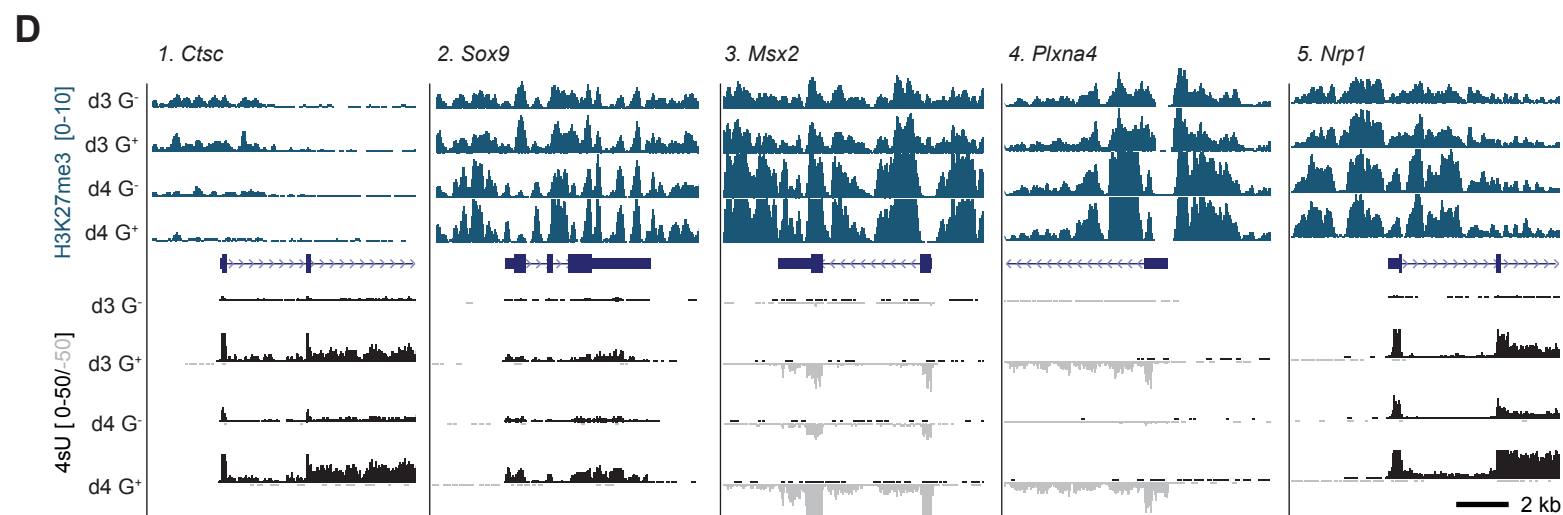

Supplement: S7 Fig — A) Genome browser tracks of ChIP-seq signal for H3K27me3 (upper panel) and 4sU-seq (lower panel) from the d3 G+ population at the Gsc gene loci (annotated as per the mm9 genome assembly). The 4sU-seq signal is coloured according to the transcribed strand (positive—black and negative—grey). Normalised read depths are indicated in parenthesis for each data type. Tracks represent a single matched experiment or combined replicates (n = 2) for 4sU-seq and ChIP-seq respectively. B) Scatter plots of all gene TSSs that are upregulated in G+ vs G- populations for both D3 and D4 of differentiation (Up; in red) and the subset of TSSs that are in the upper 50% of H3K27me3 ChIP-seq and 4sU-seq signal (4sU+K27+Up; red with black circles) from the d3 G+ population. Plots represent (in order from top to bottom) the H3K27m3 ChIP-seq signal (TSS +/- 500 bp from the d3 G+ population), 4sU-seq signal (from the d3 G+ population) and the ratio of H3K27me3 ChIP-seq signal (TSS +/- 500 bp) between G+ and G- populations at day 3 and 4 of differentiation. 4sU-seq data and ChIP-seq data represent the mean of three and two independent replicate experiments respectively. C) Boxplot of log2 H3K27me3 ratios (G+/G-) for all upregulated and 4sU+K27+ upregulated gene sets at day 3 and day 4 of differentiation (number of genes based on gene symbol annotation). Significant changes between the populations for each gene set are indicated above their respective plot (> 0.05n/s, p≤0.05* and p≤0.01**; determined using a Wilcoxon signed rank test). ChIP-seq data represent the mean of two independent replicate experiments. D) Genome browser tracks of ChIP-seq signal for H3K27me3 (upper panel) and 4sU-seq (lower panel) for the genes indicated in panel (B; 1–5). Tracks are displayed as in panel (A). (PDF) [file pgen.1011584.s007.pdf]
