# Supplementary material for: PRC2 promotes canalisation during endodermal differentiation
Source: PLoS Genet. 2025 Jan 30;21(1):e1011584. doi: 10.1371/journal.pgen.1011584 (PMC11813121; doi:10.1371/journal.pgen.1011584)

A

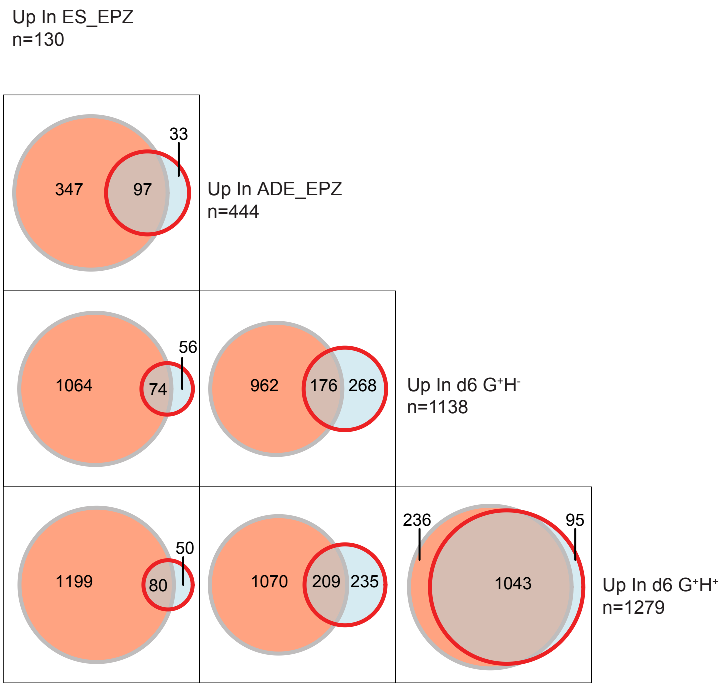

B

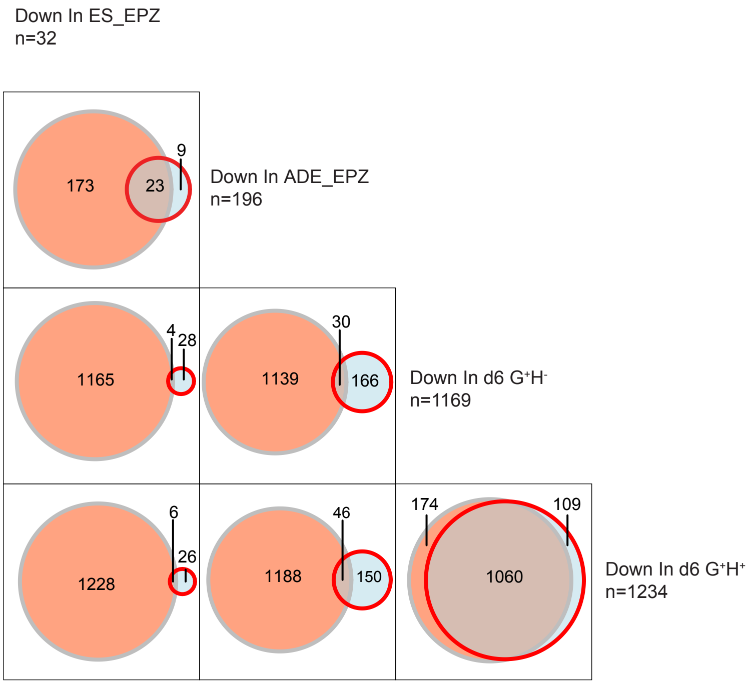

C

Rank	Term	GO ID
1	developmental process	GO:0032502
2	anatomical structure development	GO:0048856
3	multicellular organism development	GO:0007275
4	animal organ development	GO:0048513
5	anatomical structure morphogenesis	GO:0009653
6	system development	GO:0048731
7	biological regulation	GO:0065007
8	nervous system development	GO:0007399
9	cell differentiation	GO:0030154
10	multicellular organismal process	GO:0032501
11	cellular developmental process	GO:0048869
12	nitric oxide mediated signal transduction	GO:0007263
13	neuron differentiation	GO:0030182
14	embryonic placenta development	GO:0001892
15	negative regulation of cellular process	GO:0048523
16	cell development	GO:0048468
17	epithelial cell development	GO:0002064
18	embryonic organ development	GO:0048568
19	generation of neurons	GO:0048699
20	cell morphogenesis	GO:0000902

D

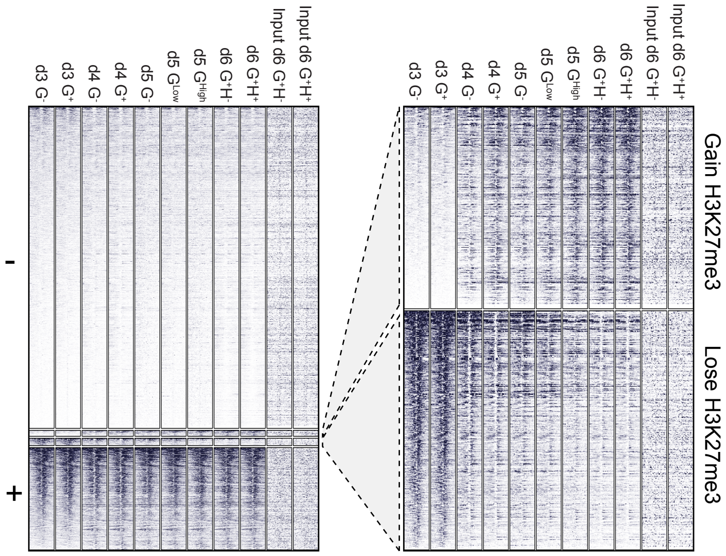

Supplement: S8 Fig — A & B) Venn diagram comparing the overlap between differentially expressed gene (DEG) sets. Overlap for up and down-regulated DEGs are shown for; ADE d6 G+H+ vs ESCs, ADE d6 G+H- vs ESCs, ES_EPZ treated ADE d6 vs control treated ADE d6, and ADE_EPZ treated ADE d6 vs control treated ADE d6. C) The top 20 significantly enriched biological processes terms from a GProfiler GO analysis performed on the genes set that were upregulated upon EPZ treatment but not in ADE d6 vs ESCs (all displayed results have an adjusted p.value < 0.05). D) Heatmap of H3K27me3 ChIP-seq signal at TSSs (+/- 5 kb) subdivided into genes with no/low H3K27me3 levels (‘-’), gain H3K27me3 levels, lose H3K27me3 levels or consistent positive H3K27me3 levels (‘+’) throughout the ADE differentiation for the indicated samples. (PDF) [file pgen.1011584.s008.pdf]
