# Supplementary material for: PRC2 promotes canalisation during endodermal differentiation
Source: PLoS Genet. 2025 Jan 30;21(1):e1011584. doi: 10.1371/journal.pgen.1011584 (PMC11813121; doi:10.1371/journal.pgen.1011584)
Supplement: Attachment — Submitted filename: Reponse to reviewers.pdf [file pgen.1011584.s018.pdf]

THE UNIVERSITY of EDINBURGH
Institute for Regeneration and Repair

Centre for Regenerative Medicine

Institute for Regeneration and Repair
The University of Edinburgh
Edinburgh BioQuarter
5 Little France Drive
Edinburgh
EH16 4UU

Tel: +44 (0) 131 651 9500
Fax: +44 (0) 131 651 9501

regenerative-medicine@ed.ac.uk
www.ed.ac.uk/regenerative-medicine

5th December 2024

Re. Response to reviewers - PGENETICS-D-24-00986

Dear reviewers and PLOS Genetics editorial team,

We thank the reviewers for their supportive and constructive feedback. We believe that the points raised by the reviewers have been addressed in the revised version of the manuscript as outlined below and have helped to strengthen and improve the study. The key finding relating to the temporal loss of H3K27me3 relative to transcriptional activation is an important one for the field (as the reviewers kindly point out). We have added additional analysis here which, in combination with more data, will hopefully satisfy the reviewers that our cell population-based conclusions hold true. All additional analysis and/or clarifications have been updated, not only in the body text but also in the materials and methods and figure legends. Please find our point-by-point responses to the comments below.

On behalf of myself, Joshua Brickman and the other authors, I thank you for your consideration.

Yours sincerely,

Dr Rob Illingworth - Group Leader. Centre for Regenerative Medicine

Reviewer #1 (Evidence, reproducibility and clarity (Required)):
Summary:
To explore the relationship between histone post-translational modifications (H3K4me3 and H3K27me3) and enhancer activation with gene expression during early embryonic development, the authors used a monolayer differentiation approach to convert mouse embryonic stem cells (ESCs) into Anterior Definitive Endoderm (ADE). They monitored differentiation stages using a dual reporter mESC line (B6), which has fluorescent reporters inserted at the Gsc (GFP) and Hhex (Redstar) loci. Their analyses indicate that the differentiating cells advanced through stages similar to those in the embryo, successfully

converting into endoderm and ADE with high efficiency. This is elegant and well performed stem cell biology.

Their subsequent genome-wide and nascent transcription analyses confirmed that the in vitro gene expression changes correlated with developmental stages and confirmed that transcriptional activation precedes mRNA accumulation. They then focussed on linking active enhancers and histone modifications (H3K4me3 and H3K27me3) were with gene expression dynamics. Finally, the performed PRC2 inhibition and showed that, while it enhanced differentiation efficiency, it also induced ectopic expression of non-lineage specific genes.

Major comments:
In terms of mechanistic advances, they propose that transcriptional up-regulation does not require prior loss of H3K27me3, which they show appears to lag behind gene activation, but critically, on a likely mixed population level. I am sceptical of their interpretation of their data because they are looking at heterogenous populations of cells. To explain, one could imagine a particular H3K27me3 coated gene that gets activated during differentiation. In a population of differentiating cells, while the major sub-population of cells could retain H3K27me3 on this particular gene when it is repressed, a minority sub-population of cells could have no H3K27me3 on the gene when it is actively transcribed. The ChIP and RNA-seq results in this mixed cell scenario would give the wrong impression that the gene is active while retaining H3K27me3, when in reality, it's much more likely that the gene is never expressed when its locus in enriched with the repressive H3K27me3 modification. Therefore, to support their claim, they would have to show that a particular gene is active when its locus is coated with H3K27me3. Personally, I don't feel this approach would be worth pursuing.

They also report that inhibition of PRC2 using EZH2 inhibitor (EPZ6438) enhanced endoderm differentiation efficiency but led to ectopic expression of pluripotency and non-lineage genes. However, this is not surprising considering the established role of Polycomb proteins as repressors of lineage genes.

>Author Response

*We agree with the point regarding the delayed loss of H3K27me3 relative to gene activation, and indeed this same point has been raised by reviewer 3 (see below). Our cell-population based data does not allow us to directly test if gene up-regulation in a small population of cells from TSSs lacking H3K27me3, accounts for the observed result. Furthermore, there are currently no robust methods to determine cell- or allele-specific expression simultaneously with ChIP/Cut and Run for chromatin marks. However, we have included the following additional text (and associated data in new supplemental material - **S7 Fig**) that we believe strongly supports our conclusions.*

'However, it is possible that H3K27me3 levels were invariant because i). activation occurred primarily at genes with little or no H3K27me3 at their TSS or ii). heterogeneous transcriptional activation from only a small fraction of the cells within the sorted population. Our FACs isolation strategy used to prepare cell populations for ChIP, microarray expression and 4sU-seq analysis was based on expression (or lack thereof) of the fluorescent GSC-GFP reporter (**Fig 1** and **S2A**). This means that every cell in each G^+ population expressed the GSC fluorescent reporter (at least at the protein level) at the point of isolation. This activation occurred despite the presence of appreciable and invariant levels of H3K27me3 at the TSS of the *Gsc* gene in both G^+ and G^- populations at day 3 of differentiation (**S7A Fig**). H3K27me3 levels at the *Gsc* gene were then substantially reduced in the G^+ relative to the G^- populations at day 4 comparable to what was observed for all upregulated genes (**Fig 5**, **S6** and **S7A**). As *Gsc* was activated in all G^+ cells despite high levels of H3K27me3, this argued against low H3K27me3 or heterogenous expression as an explanation. However, it is possible that *Gsc*

represented an isolated example, and so we tested this more generally. We selected upregulated TSSs that had a greater than median value for both transcription (4sU-seq) and H3K27me3 in the d3 G^+ population (n=35 of 125 genes; **S7B Fig**). Both combined and candidate-based analysis of these genes showed that transcriptional activation occurred in the presence of substantial and invariant levels of H3K27me3 at day 3 followed by a subsequent reduction by day 4 of differentiation (**S7B – S7D Fig**). Importantly, many of these genes yielded high absolute 4sU-seq signal, comparable to that observed for *Gsc* (**S7A Fig**), arguing against transcriptional activation being limited to only a small subpopulation of cells. Taken together these findings suggest that transcriptional up-regulation does not require prior loss of H3K27me3, and that H3K27me3 loss lags behind transcriptional activation, at least for a subset of genes during the early stages of ADE.*'*

Reviewer #2 (Evidence, reproducibility and clarity (Required)):
In this paper the authors profile gene expression, including active transcription, and histone modifications (k4 and k27me3) during a complex differentiation protocol from ES cells, which takes advantage of FACS sorting of appropriate fluorescent reporters. The data is of good quality and the experiments are well performed. The main conclusion, that the analyzed histone marks channel differentiation more than they directly allow/block it, is well supported by the data. The paper is interesting and will represent a good addition to an already extensive literature. I have however a few major concerns, described below:

1/ K4me3 may show more changes than they interpret, at least over the +1 nucl. An alternative quantification to aggregate profiles should be used to more directly address the questions regarding the correlations between histone mods and gene expression.

>Author Response

Whilst we state that H3K4me3 levels are somewhat less dynamic with resepct to gene expression relative to H3K27me3, quantification of individual TSS (+/- 500 bp) did show a direct correlation with gene expression (Fig 5 and S6). To further explore this in response to the reviewer's comment we quantified H3K4me3 and H3K27me3 signal at different windows around the TSS to see if this identified a more substantial shift in the H3K4me3 signal relative to gene expression than was evident in the averaged profiles (this included a 300 bp / ~2 nucleosome window immediately 3' of the TSS; see reviewer figure below). This showed that the magnitude of dynamic changes in both modifications were largely unaffected by the window size being quantified/analysed. However, as noted by yourself and reviewer three, the meta summary profiles were not fit for purpose and along with the other changes to Fig 4 have now been replaced with boxplots of ChIP-seq signal that better represent each sample and the data distribution across the genes in that sample (Fig 4D).

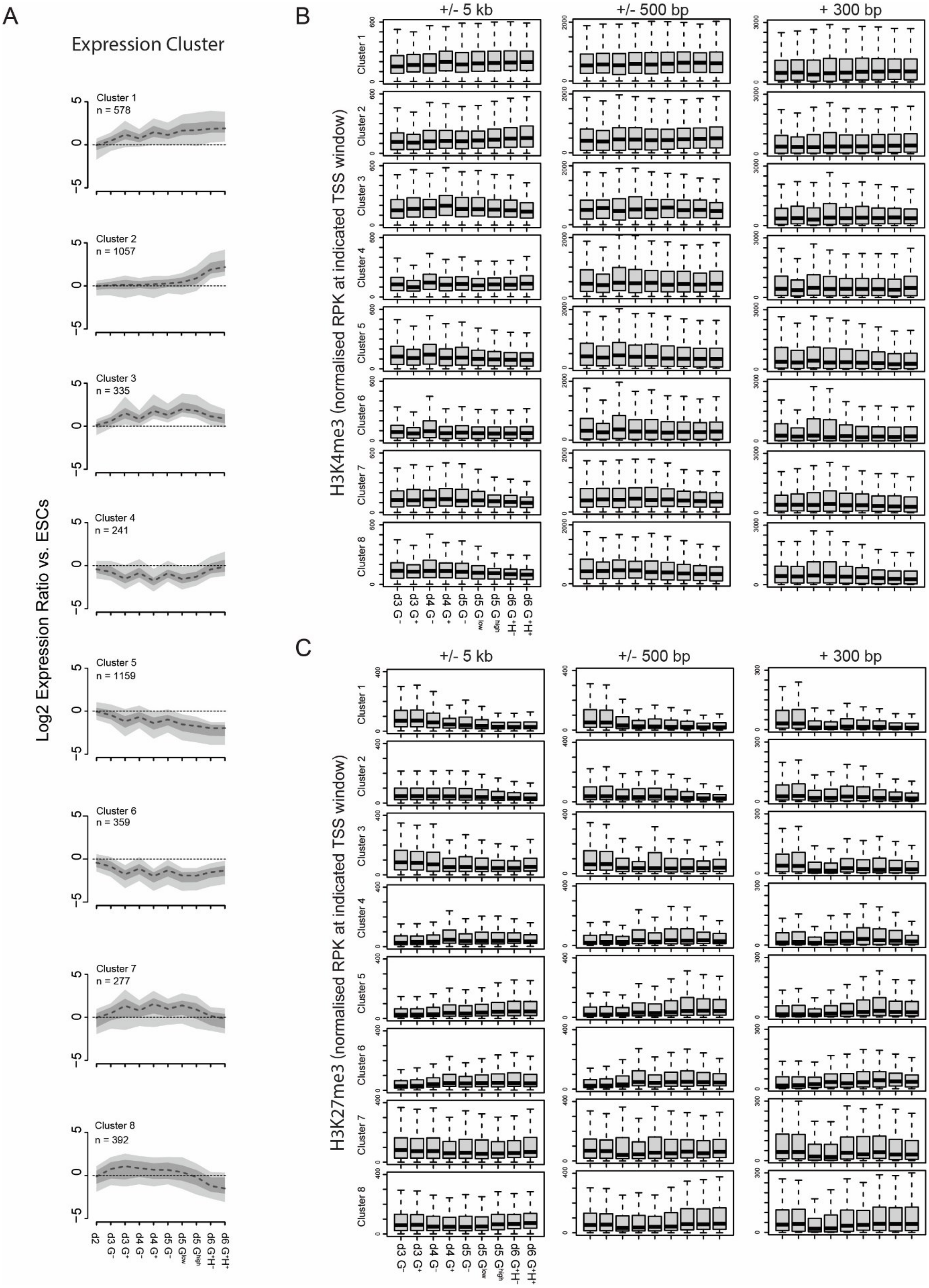

2/ Related to the previous point, it appears clear in Fig.4 that the promoters of each gene expression cluster do not belong to a single chromatin configuration. I think it would be important to:

1/ cluster the genes based on promoter histone mods and interrogate gene expression and

cluster allocation (basically the reverse to what is presented)
2/ order the genes in the heatmaps identically for K4me3 and K27me3 to more easily understand the respective chromatin composition per cluster

>Author Response

We thank the reviewer for these suggestions. We have revised Fig4 so that the ChIP data in each expression cluster is clustered based on the K4me3 and K27me3 signal and that the genes in both datasets are ordered the same based on this clustering. We agree that this is a less biased approach to present the data and is visually clearer also (Fig 4C). In addition, we have re-analysed the data and clustered it based on the ChIP-data alone independent of gene expression. This analysis showed a clear categorisation of the genes based on the modification state that identified functionally distinct sets of genes with distinct promoter type tendencies (e.g. +/- CGIs). See new S5 Fig panels C-E and the new additional text which refers to it is as follows.

'This analysis necessarily focussed on the relatively small fraction of genes whose expression changed significantly during the course of the differentiation (~18% or 3,878 of the 22,040 Refseq genes analysed; **Fig 4C-D**). However, to determine if chromatin modification state could stratify all genes independently of gene expression, we performed the reciprocal analysis by clustering TSSs based on H3K4me3 and H3K27me3 levels. The resulting gene sets were distinct with respect to promoter sequence composition (+/- CGIs), gene expression dynamics and importantly, functional classification (**S5C-E Fig, S5 Table** and **S6 Table).** Whilst dynamic chromatin profiles identified gene sets with variable expression levels (e.g. clusters 1, 4 and 8); constitutively low/low (cluster 2), low/high (cluster 10), high/low (cluster 5) or high/high (cluster 9) levels of H3K4me3 and H3K27me3 enriched for genes associated with immune, neuroectodermal, housekeeping and broad-developmental functions respectively (**S5C-E Fig, S5 Table** and **S6 Table**). This demonstrated that H3K4me3 and H3K27me3 levels could effectively stratify genes independently of their expression status during ADE differentiation.'

3/ Also, as it is apparent that not all promoters in every cluster are enriched for the studied marks, could the authors separately analyze these genes? What are they? Do they use alternative promoters?

>Author Response

Indeed, this is the case. Whilst there is significant enrichment of H3K27me3 at the TSS of developmentally regulated genes, not all genes whose expression changes during the differentiation are polycomb (PRC2) targets. As discussed above we now stratify the expression clusters based on the ChIP-signal and also include the additional ChIP-based clusters data (S5 Fig). The ChIP-defined clusters, in particular clusters 2 and 3 that have relatively low H3K4me3 and H3K27me3 levels have a reduced proportion of genes with CGI promoters. This is indicated in S5 Fig C in parenthesis and the expression and functional enrichment of these clusters is shown in S5C-E.

4/ The use of 4SU-seq to identify active enhancers is welcome; however, I have doubts it is working very efficiently: for instance, in the snapshots shown in Fig.2A, the very active Oct4 enhancers in ES cells are not apparent at all... More validation of the efficiency of the approach seems required.

>Author Response

*The 4sU-seq data shown in **Fig 2A** was generated in samples isolated from day 3 and 4 of the ADE differentiation. It is therefore likely that the enhancers have been partly or wholly decommissioned at this point as differentiation progressed. Indeed, in a separate study we*

generated 4sU-seq data using the same protocol/conditions/read depths as presented here but in ES cells and differentiated NPCs (day 3 to 7). Indeed transcription is identified at Pou5f1/Oct 4 *enhancers in ESCs (arrowed in the screenshot below) which are extinguished upon differentiation to neural progenitor cells (NPCs); data take from PMID: 31494034). Please see example browser track that shows the existence of such 'eRNAs' at enhancer proximal to the* Pou5f1 *locus in our ESC data but that become extinguished upon differentiation into NPCs.*

Oct4 Locus

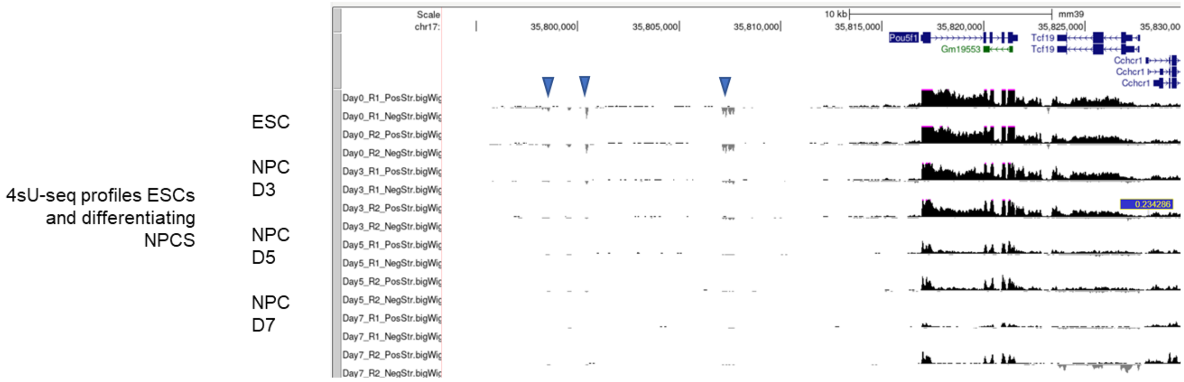

NB. All tracks are scaled the same within each datatype

5/ The effects of the EZH2 inhibitor are quite minor regarding the efficiency of the differentiation as analyzed by FACS, despite significant gene expression changes. To the knowledge of this referee, this is at odds with results obtained with Ezh2 ko ES cells that display defects in mesoderm and endoderm differentiation. I have issues reconciling these results (uncited PMID: 19026780). Either the authors perform more robust assays (inducible KOs) or they more directly explain the limitations of the study and the controversies with published work.

>Author Response

We agree that this result appears to be at odds with the findings in (PMID: 19026780). This is likely due to the fact that we are acutely reducing H3K27me3 levels for a short period either during or immediately preceding the differentiation rather than removing PRC2 constitutively / genetically. Furthermore, we use a defined regiment of cytokines to direct differentiation towards ADE, whereas this study (PMID: 19026780) used a spontaneous differentiation paradigm (LIF withdrawal) which may be more susceptible to H3K27me3 / PRC2 disruption.

We included the following new text in the discussion to address this point.

'These findings are however at odds with previous work that found that reduced H3K27me3 deposition caused impaired mesendodermal differentiation [68]. Whilst the reason for this discrepancy is unknown, the conclusions from this study were based on constitutive genetic PRC2 ablation rather than short-term acute PRC2 inhibition. The elevated levels of mesendodermal gene expression observed in PRC2 deficient ESCs could have directly impaired differentiation and/or reduced the relative fold transcriptional activation observed. More likely however, is the fact that this study uses an un-constrained differentiation paradigm based on LIF withdrawal, whereas here we employ a defined regimen of cytokines to direct endodermal differentiation [68].'

Director: Professor Stuart J Forbes MBChB PhD FRCP(Ed) FRSE FMedSci
The University of Edinburgh is a charitable body, registered in Scotland, with registration number SC005336

Minor
1/ please add variance captured to PCA plots

> *This has been added to the plot and indicated in the legends of Fig1 and 6.*

2/ Fig1E add color scales to all heatmaps

> *All existing and new heatmaps now have colour bars.*
3/ Fig4C,D are almost impossible to follow, please find a way to identify better the clusters/samples and make easier to correlate all the variables

> *This was unclear and has now been replaced using the boxplot representation outlined above.*

Reviewer #3 (Evidence, reproducibility and clarity (Required)):
This study investigates the role of chromatin-based regulation during cell fate specification. The authors use an ESC model of differentiation into anterior primitive streak and subsequently definitive endoderm, which they traced via a dual-reporter system that combines GSC-GFP and HHEX-RedStar. The authors mapped changes in (nascent) gene expression and histone modifications (H3K4me3/H3K27me3) at key timepoints and within different populations over six days of differentiation. Finally, the authors test the functional implications of H3K27me3 landscapes via PRC2 inhibition.

The majority of data chart the descriptive changes in (epi)genomic and transcriptional dynamics coincident with cell differentiation. The use of nascent transcriptomics improves the temporal resolution of expression dynamics, and is an important strategy. By and large the data reinforce established paradigms. For example, that transcription is the dominant mechanism regulating mRNA levels, or that dynamic chromatin states changes occur and largely corelate of gene activity. They also identify putative enhancers with profiling data, albeit these are not validated, and confirm that PRC2 inhibition impacts cell fate processes - in this case promoting endodermal differentiation efficiency. Overall, the study is relatively well-performed and clearly written, with the omics profiling adding more datasets from in vitro cell types that can be difficult to characterise in vivo. Whilst the majority of the study may be considered incremental, the key finding is the authors conclusion that H3K27me3 is subordinate to gene activity rather than an instructive repressor. If borne out, this would mark an important observation with broad implications. However, in my view this conclusion is subject to many confounders and alternative interpretations, and the authors have not ruled out other explanations. Given the centrality of this to the novelty of the study, I would encourage further analysis/stratification of existing data, and potentially further experiments to provide more confidence in this key conclusion.

Primary issue
1.) The authors show that at the earliest timepoint (d3), nascent gene activation of a handful of genes between G+ and G- populations is not associated with a FC loss of H3K27me3. From this the authors extrapolate their key conclusion that H3K27me3 is subordinate. Causality of chromatin modifications in gene regulation is critical to decipher, and therefore this is an important observation to confirm. Below I go through the possible confounders and issues with the conclusion at this point.

(i) Single-cell penetrance. A possible (likely?) possibility is that gene activation initially occurs in a relatively small subset of cells at d3. Because these genes are expressed lowly prior to this, they will register as a significant upregulation in bulk analysis. However, in this scenario H3K27me3 would only be lost from a small fraction of cells, which would not be detectable against a backdrop of most cells retaining the mark. In short, the authors have not ruled out

heterogeneity driving the effect. Given the different dynamic range of mRNA and chromatin marks, and that a small gain from nothing (RNA) is easier to detect than a small loss from a pre-marked state (chromatin), investigating this further is critical to draw the conclusions the authors have.

(ii) Initial H3K27me3 levels. The plots in Fig 5 show the intersect FC of H3K27me3 and gene expression. Genes that activate at d3 show no loss of H3K27me3. However, it is important to characterise (and quantitate) whether these genes are significantly marked by H3K27me3 in the first place, which I could not find in the manuscript. Many/several of the genes may not be polycomb marked or may have low levels to begin with. This would obviously confound the analysis, since an absence/low K27 cannot be significantly lost and is unlikely to be functional. Thus, the DEG geneset should be further stratified into H3K27me3+ and K27me3- promoter groups/bins, with significance and conclusions based on the former only (e.g. boxplot in 5F).

(iii) Sample size. The conclusions are based on a relatively small number of genes that upregulate between G+ and G- (n=55 in figure by my count, text mentions n=52). Irrespective of the other confounders above, this is quite a small subset to make the sweeping general conclusion that "loss of the repressive polycomb mark H3K27me3 is delayed relative to transcriptional activation" in the abstract. Indeed, the small number of DEG suggests the cell types being compared are similar and perhaps therefore have specific genomic features (this could be looked at) that drive .

>Author Response

*These are very good points and are also raised by reviewer 1 (see above). We have one example where we can definitively interrogate single cell protein expression, in our current data. Gsc (as monitored by GSC-GFP FACS and the bulk RNA analysis) meets the criteria of being robustly upregulated in all FACs sorted cells in the presence of high levels of H3K27me3 in the D3G+ population. We believe that the additional analysis (**S7 Fig**) and the associated text addresses the reviewer's concerns about both the levels of expression and magnitude of H3K27me3. With respect to the third point, the numbers are low (although here I present data from the 4SU analysis with approximately three times more data points) however, the point here is not too say this happens in every instance of gene activation but more that it can happen and not just at a small subset of outlier genes. This is important, as the reviewer notes, in our understanding of how polycomb repression is relieved during development. The following text has been added that relates to the additional data that has been included.*

'However, it is possible that H3K27me3 levels were invariant because i). activation occurred primarily at genes with little or no H3K27me3 at their TSS or ii). heterogeneous transcriptional activation from only a small fraction of the cells within the sorted population. Our FACs isolation strategy used to prepare cell populations for ChIP, microarray expression and 4sU-seq analysis was based on expression (or lack thereof) of the fluorescent GSC-GFP reporter (**Fig 1** and **S2A**). This means that every cell in each G^+ population expressed the GSC fluorescent reporter (at least at the protein level) at the point of isolation. This activation occurred despite the presence of appreciable and invariant levels of H3K27me3 at the TSS of the *Gsc* gene in both G^+ and G^- populations at day 3 of differentiation (**S7A Fig**). H3K27me3 levels at the *Gsc* gene were then substantially reduced in the G^+ relative to the G^- populations at day 4 comparable to what was observed for all upregulated genes (**Fig 5**, **S6** and **S7A**). As *Gsc* was activated in all G^+ cells despite high levels of H3K27me3, this argued against low H3K27me3 or heterogenous expression as an explanation. However, it is possible that *Gsc* represented an isolated example, and so we tested this more generally. We selected upregulated TSSs that had a greater than median value for both transcription (4sU-seq) and H3K27me3 in the d3 G^+ population (n=35 of 125 genes; **S7B Fig**). Both combined and candidate-based analysis of these genes showed that transcriptional activation occurred in the presence of substantial and invariant levels of H3K27me3 at day 3

followed by a subsequent reduction by day 4 of differentiation (**S7B – S7D Fig**). Importantly, many of these genes yielded high absolute 4sU-seq signal, comparable to that observed for *Gsc* (**S7A Fig**), arguing against transcriptional activation being limited to only a small subpopulation of cells. Taken together these findings suggest that transcriptional up-regulation does not require prior loss of H3K27me3, and that H3K27me3 loss lags behind transcriptional activation, at least for a subset of genes during the early stages of ADE.'

The reviewer points out that the number of genes varies a little between analyses. This arises due to the annotation of multiple TSSs per genes in some cases (plots show values for different transcripts whereas the numbers represent genes – either based on gene symbol or refseq annotation). I have added text throughout the legends to better define what type of annotation that is being referred to.

With respect to this being a rare phenomenon, we mean to say that invariant H3K27me3 is an observed phenomenon that can occur at genes, rather than to say it is always or even the majority case. This being said, the new S7 Fig that ranks based on H3K27e3 levels does demonstrate that this phenomenon holds true for an appreciable fraction of differentially expressed genes and is not restricted to Gsc *alone. We add the following statement to the results section to qualify that this phenomenon occurs in a subset of genes and during the ADE differentiation as we cannot conclude that this is also true in other cases (although unpublished synthetic experiments ongoing in the Illingworth lab suggests that this is likely not a special case).*

'Taken together these findings suggest that transcriptional up-regulation does not require prior loss of H3K27me3, and that H3K27me3 loss lags behind transcriptional activation, at least for a subset of genes during the early stages of ADE.'

Other comments:
2.) The authors show that promoter H3K4me3 corelates well with gene expression dynamics in their model. They conclude that "transcription itself is required for H3K4me3 deposition", or in other words is subordinate. This may well be the case but from their correlative data this cannot be inferred. Indeed, several recent and past papers have shown that H3K4me3 itself can directly modulate transcription, for example by triggering RNA II pause-release, by preventing epigenetic silencing and/or by recruiting the PIC. The authors could point out or discuss these alternative possibilities to provide a more balanced discourse.

>Author Response

We agree and we have altered the statement in the discussion to reflect this point (and associated references) as follows.

'This is consistent with studies demonstrating that H3K4me3 deposition and transcription are intimately and reciprocally coupled [54-58].'

3.) The labelling of some figures is unclear. In Fig 4C and 4D (right) it is impossible to tell what sample each of the lines represents. It is also not clear what the blue zone corresponds to in genome view plots (the whole gene?). Moreover, the replicate numbers are not shown in figure legends.

>Author Response

We agree that the data presented in 4C and D was unclear in the original manuscript. We have now swapped this representation for a more substantial and informative boxplot representation in a modified Fig 4 (additional analysis is also presented in S5 Fig)

The blue shaded area represented the full extent of the key genes being discussed in the screen shot, this was mentioned in the legend. However, on re-examining this we decided to remove this as it was distracting (this is true throughout the manuscript).

Replication is now documented in the legends throughout the manuscript.

4.) It would be nice to provide more discussion to reconcile the conclusions that H3K27me3 in endoderm differentiation is subordinate and the final figure showing inhibiting H3K27me3 has a significant effect on differentiation, since the latter is the functional assessment.

>Author Response

We hope that this point is clearer now in the discussion text.

'While our findings corroborate the idea that PRCs acts to resist transcription to ensure the appropriate gene expression landscape is established during developmental programmes, our data do not support an instructive role for PRCs in establishing the transcriptional landscape of specific cell types.'
