# Supplementary material for: PRC2 promotes canalisation during endodermal differentiation
Source: PLoS Genet. 2025 Jan 30;21(1):e1011584. doi: 10.1371/journal.pgen.1011584 (PMC11813121; doi:10.1371/journal.pgen.1011584)
Supplement: Attachment — Submitted filename: 146566_1_rebuttal_3120161_sxxsjx.docx [file pgen.1011584.s017.docx]

**Manuscript number:** RC-2024-02484

**Corresponding author(s):** Illingworth, Robert S and Brickman, Joshua M

## 1. General Statements

We thank the reviewers for their supportive and constructive feedback. All points raised will be addressed as outlined below in a revised version of the manuscript. The key finding relating to the temporal loss of H3K27me3 relative to transcriptional activation is an important one for the field (as the reviewers kindly point out). We have added additional analysis here which, in combination with a more data as outlined below, will hopefully satisfy the reviewers that our cell population-based conclusions hold true.

## 2. Description of the planned revisions

**Reviewer #1 (Evidence, reproducibility and clarity (Required)):**
Summary:
To explore the relationship between histone post-translational modifications (H3K4me3 and H3K27me3) and enhancer activation with gene expression during early embryonic development, the authors used a monolayer differentiation approach to convert mouse embryonic stem cells (ESCs) into Anterior Definitive Endoderm (ADE). They monitored differentiation stages using a dual reporter mESC line (B6), which has fluorescent reporters inserted at the Gsc (GFP) and Hhex (Redstar) loci. Their analyses indicate that the differentiating cells advanced through stages similar to those in the embryo, successfully converting into endoderm and ADE with high efficiency. This is elegant and well performed stem cell biology.

Their subsequent genome-wide and nascent transcription analyses confirmed that the in vitro gene expression changes correlated with developmental stages and confirmed that transcriptional activation precedes mRNA accumulation. They then focussed on linking active enhancers and histone modifications (H3K4me3 and H3K27me3) were with gene expression dynamics. Finally, the performed PRC2 inhibition and showed that, while it enhanced differentiation efficiency, it also induced ectopic expression of non-lineage specific genes.

Major comments:
In terms of mechanistic advances, they propose that transcriptional up-regulation does not require prior loss of H3K27me3, which they show appears to lag behind gene activation, but critically, on a likely mixed population level. I am sceptical of their interpretation of their data because they are looking at heterogenous populations of cells. To explain, one could imagine a particular H3K27me3 coated gene that gets activated during differentiation. In a population of differentiating cells, while the major sub-population of cells could retain H3K27me3 on this particular gene when it is repressed, a minority sub-population of cells could have no H3K27me3 on the gene when it is actively transcribed. The ChIP and RNA-seq results in this mixed cell scenario would give the wrong impression that the gene is active while retaining H3K27me3, when in reality, it's much more likely that the gene is never expressed when its locus in enriched with the repressive H3K27me3 modification. Therefore, to support their claim, they would have to show that a particular gene is active when its locus is coated with H3K27me3. Personally, I don't feel this approach would be worth pursuing.

They also report that inhibition of PRC2 using EZH2 inhibitor (EPZ6438) enhanced endoderm differentiation efficiency but led to ectopic expression of pluripotency and non-lineage genes. However, this is not surprising considering the established role of Polycomb proteins as repressors of lineage genes.

Reviewer #1 (Significance (Required)):
I feel that this is a solid and well conducted study in which the authors model early development in vitro. It should be of interest to researchers with an interest in more sophisticated in vitro differentiation systems, perhaps to knockout their gene of interest and study the consequences. However, I don't see any major mechanistic advances in this work.

*>Author Response*

*We agree with the point regarding the delayed loss of H3K27me3 relative to gene activation, and indeed this same point has been raised by reviewer 3 (see below). Our cell-population based data does not allow us to directly test if gene up-regulation in a small population of cells from TSSs lacking H3K27me3, accounts for the observed result. Furthermore, there are currently no robust methods to determine cell- or allele-specific expression simultaneously with ChIP/Cut and Run for chromatin marks. However, we provide the following additional evidence that strongly supports our conclusions.*

*Our FACs isolation strategy used to prepare cell populations for ChIP, microarray expression and 4sU-seq analysis is based on expression (or lack thereof) of a fluorescent* GSC-GFP *reporter. This means that every cell in the G+ populations express the Gsc fluorescent reporter, at least at the protein level, at the point of isolation. This is despite the presence of appreciable and invariant levels of H3K27me3 at the TSS of the Gsc gene in both G+ and G- populations at day 3 of differentiation. Comparable to our meta-analysis of all upregulated genes shown in the original manuscript (****Figure 5*** *and* ***S5****), H3K27me3 levels are then subsequently reduced in the G+ relative to the G- populations at day 4. The transcriptional changes which correspond to the GSG-GFP reporter expression and associated ChIP-seq data are shown in the reviewer figure below (****Fig R1 A)****. To further support our observations, we sought to rule out the possibility that the shift in H3K27me3 and transcription were from mutually exclusive gene sets, from nominal transcription levels or from sites with low level H3K27me3. To do this with a gene set of sufficient size to yield a robust result, we selected upregulated TSSs that had a greater than median value for both transcription (4sU-seq) and H3K27me3 (n=49 of 159 genes;* ***Fig R1 B****). Meta-analysis of these genes showed that, as for all upregulated gene TSS (n=159), transcriptional activation occurred in the presence of substantial and invariant levels of H3K27me3 at day 3 followed by a subsequent reduction by day 4 of differentiation (****Fig R1 C****). Importantly, many of these genes yielded high absolute 4sU-seq signal, comparable to that of Gsc, arguing against transcriptional activation being limited to a small subpopulation of cells.*

**Reviewer #2 (Evidence, reproducibility and clarity (Required)):**
In this paper the authors profile gene expression, including active transcription, and histone modifications (k4 and k27me3) during a complex differentiation protocol from ES cells, which takes advantage of FACS sorting of appropriate fluorescent reporters. The data is of good quality and the experiments are well performed. The main conclusion, that the analyzed histone marks channel differentiation more than they directly allow/block it, is well supported by the data. The paper is interesting and will represent a good addition to an already extensive literature. I have however a few major concerns, described below:

1/ K4me3 may show more changes than they interpret, at least over the +1 nucl. An alternative quantification to aggregate profiles should be used to more directly address the questions regarding the correlations between histone mods and gene expression.

*>Author Response*

*Whilst we state that H3K4me3 levels are somewhat invariant at differentially expressed genes relative to H3K27me3, quantification of individual TSS (+/- 500 bp) did show a direct correlation with gene expression (Figure 5 and S5). To further explore this in response to the reviewer’s comment we will quantify K4me3 signal at the +1 nucleosome to determine if this yields more substantial differences than that observed more broadly across TSSs.*

2/ Related to the previous point, it appears clear in Fig.4 that the promoters of each gene expression cluster do not belong to a single chromatin configuration. I think it would be important to:
1/ cluster the genes based on promoter histone mods and interrogate gene expression and cluster allocation (basically the reverse to what is presented)
2/ order the genes in the heatmaps identically for K4me3 and K27me3 to more easily understand the respective chromatin composition per cluster

*>Author Response*

*We thank the reviewer for these suggestions and will include these analyses in a revised manuscript.*

3/ Also, as it is apparent that not all promoters in every cluster are enriched for the studied marks, could the authors separately analyze these genes? What are they? Do they use alternative promoters?

*>Author Response*

*Indeed, this is the case. Whilst there is significant enrichment of H3K27me3 at the TSS of developmentally regulated genes, not all genes whose expression changes during the differentiation will be polycomb targets. We will further stratify these clusters as suggested and determine what distinguishes the subsets. If informative, this data will be included in a revised manuscript.*

4/ The use of 4SU-seq to identify active enhancers is welcome; however, I have doubts it is working very efficiently: for instance, in the snapshots shown in Fig.2A, the very active Oct4 enhancers in ES cells are not apparent at all... More validation of the efficiency of the approach seems required.

*>Author Response*

*The 4sU-seq data shown in* ***Figure 2A*** *was generated in samples isolated from day 3 and 4 of the ADE differentiation. It is therefore likely that the enhancers have been partly or wholly decommissioned at this point. Indeed, in a separate study we generated 4sU-seq data using the same protocol and conditions as presented here but in ES cells and differentiated NPCs (day 3 to 7) and indeed see transcription at Oct 4 enhancers in ESCs (arrowed in the screenshot below) which are extinguished upon differentiation to neural progenitor cells (NPCs); data from PMID: 31494034).*

5/ The effects of the EZH2 inhibitor are quite minor regarding the efficiency of the differentiation as analyzed by FACS, despite significant gene expression changes. To the knowledge of this referee, this is at odds with results obtained with Ezh2 ko ES cells that display defects in mesoderm and endoderm differentiation. I have issues reconciling these results (uncited PMID: 19026780). Either the authors perform more robust assays (inducible KOs) or they more directly explain the limitations of the study and the controversies with published work.

*>Author Response*

*We agree that this result appears to be at odds with the findings in (PMID: 19026780). This is likely due to the fact that we are acutely reducing H3K27me3 levels for a short period either during or immediately preceding the differentiation rather than removing PRC2 function genetically. This, likely provides a less pronounced defect on the ability to generate endodermal cells. However, we cannot address this without further experimentation which is beyond the scope of this study. We will more fully discuss the results in the context of this and other studies and discuss the limitations of the study in this regard.*

Minor
1/ please add variance captured to PCA plots
2/ Fig1E add color scales to all heatmaps
3/ Fig4C,D are almost impossible to follow, please find a way to identify better the clusters/samples and make easier to correlate all the variables

*>Author Response*

*We will address all of these points in a revised manuscript.*

Reviewer #2 (Significance (Required)):

The paper is incremental in knowledge, and not by a big margin, as it is known already that histone mods rather channel than drive differentiation. Though, the authors do not clearly address inconsistencies with published work, especially regarding Ezh2 thought to be important to make endoderm. It is however a good addition to current knowledge, provided a better discussion of differences with published work is provided.

*>Author Response*

*As outlined above, we will address this with a more complete discussion about the distinction between the studies and what can and can’t be concluded from our approach.*

**Reviewer #3 (Evidence, reproducibility and clarity (Required)):**
This study investigates the role of chromatin-based regulation during cell fate specification. The authors use an ESC model of differentiation into anterior primitive streak and subsequently definitive endoderm, which they traced via a dual-reporter system that combines GSC-GFP and HHEX-RedStar. The authors mapped changes in (nascent) gene expression and histone modifications (H3K4me3/H3K27me3) at key timepoints and within different populations over six days of differentiation. Finally, the authors test the functional implications of H3K27me3 landscapes via PRC2 inhibition.

The majority of data chart the descriptive changes in (epi)genomic and transcriptional dynamics coincident with cell differentiation. The use of nascent transcriptomics improves the temporal resolution of expression dynamics, and is an important strategy. By and large the data reinforce established paradigms. For example, that transcription is the dominant mechanism regulating mRNA levels, or that dynamic chromatin states changes occur and largely corelate of gene activity. They also identify putative enhancers with profiling data, albeit these are not validated, and confirm that PRC2 inhibition impacts cell fate processes - in this case promoting endodermal differentiation efficiency. Overall, the study is relatively well-performed and clearly written, with the omics profiling adding more datasets from in vitro cell types that can be difficult to characterise in vivo. Whilst the majority of the study may be considered incremental, the key finding is the authors conclusion that H3K27me3 is subordinate to gene activity rather than an instructive repressor. If borne out, this would mark an important observation with broad implications. However, in my view this conclusion is subject to many confounders and alternative interpretations, and the authors have not ruled out other explanations. Given the centrality of this to the novelty of the study, I would encourage further analysis/stratification of existing data, and potentially further experiments to provide more confidence in this key conclusion.

Primary issue
1.) The authors show that at the earliest timepoint (d3), nascent gene activation of a handful of genes between G+ and G- populations is not associated with a FC loss of H3K27me3. From this the authors extrapolate their key conclusion that H3K27me3 is subordinate. Causality of chromatin modifications in gene regulation is critical to decipher, and therefore this is an important observation to confirm. Below I go through the possible confounders and issues with the conclusion at this point.

(i) Single-cell penetrance. A possible (likely?) possibility is that gene activation initially occurs in a relatively small subset of cells at d3. Because these genes are expressed lowly prior to this, they will register as a significant upregulation in bulk analysis. However, in this scenario H3K27me3 would only be lost from a small fraction of cells, which would not be detectable against a backdrop of most cells retaining the mark. In short, the authors have not ruled out heterogeneity driving the effect. Given the different dynamic range of mRNA and chromatin marks, and that a small gain from nothing (RNA) is easier to detect than a small loss from a pre-marked state (chromatin), investigating this further is critical to draw the conclusions the authors have.

(ii) Initial H3K27me3 levels. The plots in Fig 5 show the intersect FC of H3K27me3 and gene expression. Genes that activate at d3 show no loss of H3K27me3. However, it is important to characterise (and quantitate) whether these genes are significantly marked by H3K27me3 in the first place, which I could not find in the manuscript. Many/several of the genes may not be polycomb marked or may have low levels to begin with. This would obviously confound the analysis, since an absence/low K27 cannot be significantly lost and is unlikely to be functional. Thus, the DEG geneset should be further stratified into H3K27me3+ and K27me3- promoter groups/bins, with significance and conclusions based on the former only (e.g. boxplot in 5F).

(iii) Sample size. The conclusions are based on a relatively small number of genes that upregulate between G+ and G- (n=55 in figure by my count, text mentions n=52). Irrespective of the other confounders above, this is quite a small subset to make the sweeping general conclusion that "loss of the repressive polycomb mark H3K27me3 is delayed relative to transcriptional activation" in the abstract. Indeed, the small number of DEG suggests the cell types being compared are similar and perhaps therefore have specific genomic features (this could be looked at) that drive .

*>Author Response*

*These are very good points and are also raised by reviewer 1 (see above). We have one example where we can definitively interrogate single cell protein expression, in our current data. Gsc (as monitored by GSC-GFP FACS and the bulk RNA analysis) meets the criteria of being robustly upregulated in all FACs sorted cells in the presence of high levels of H3K27me3 in the D3G+ population. We believe that the additional analysis (****Figure R1A****) and the discussion above addresses the reviewer’s concerns about both the levels of expression and magnitude of H3K27me3. With respect to the third point, the numbers are low (although here I present data from the 4SU analysis with approximately three times more data points) however, the point here is not too say this happens in every instance of gene activation but more that it can happen and not just at a small subset of outlier genes. This is important, as the reviewer notes, in our understanding of how polycomb repression is relieved during development. We will also look to see if there are sequence characteristics/ motifs of these genes. In a revised manuscript we would include this data and further analysis as outlined above. The reviewer points out that the numbers vary a little between analyses. This arises due to the annotation of multiple TSSs per genes in some cases. This will be rectified throughout and made clearer in the legends.*

Other comments:
2.) The authors show that promoter H3K4me3 corelates well with gene expression dynamics in their model. They conclude that "transcription itself is required for H3K4me3 deposition", or in other words is subordinate. This may well be the case but from their correlative data this cannot be inferred. Indeed, several recent and past papers have shown that H3K4me3 itself can directly modulate transcription, for example by triggering RNA II pause-release, by preventing epigenetic silencing and/or by recruiting the PIC. The authors could point out or discuss these alternative possibilities to provide a more balanced discourse.

*>Author Response*

*We agree and this will be discussed more thoroughly and both possibilities put forward in the revised manuscript.*

3.) The labelling of some figures is unclear. In Fig 4C and 4D (right) it is impossible to tell what sample each of the lines represents. It is also not clear what the blue zone corresponds to in genome view plots (the whole gene?). Moreover, the replicate numbers are not shown in figure legends.

*>Author Response*

*We agree that the data presented in 4C and D is unclear. We will, as a minimum, collapse profiles into like populations (ESC / G- / G+ / G+H- / G+H+) which makes sense given the similarity of these populations across all analyses (see e.g. PCA analysis* ***in Figure 1****). We will also explore alternative ways of presenting the data to better highlight the dynamics and incorporate this with the changes suggested by reviewer 2. The blue shaded area represents the full extent of the key gene being discussed in the screen shot, this is mentioned in the legend but will be made clearer in a revised manuscript. Replication will also be added to the legend throughout (n=2 for ChIP-seq and n=3 for 4sU-seq).*

4.) It would be nice to provide more discussion to reconcile the conclusions that H3K27me3 in endoderm differentiation is subordinate and the final figure showing inhibiting H3K27me3 has a significant effect on differentiation, since the latter is the functional assessment.

*>Author Response*

*We will build on the points already made that suggests that whilst K27me3 is a passive repressor that serves to act against sub-threshold activating cues, it is nonetheless a critical regulator of developmental fidelity.*

Reviewer #3 (Significance (Required)):
Overall, the study's strengths are in that it characterises epigenomic dynamics within a specific and relevant cell fate model. The nascent transcriptomics adds important resolution, and underpins the core conclusions. The weakness is that data is over-interpreted at this point, and other possibilities are not adequately tested. The conclusions should therefore either be scaled back (which reduces novelty) or further analysis and/or experiments should be performed to support the conclusion. If it proves correct, this would be a significant observation for the community,

*>Author Response*

*In a revised manuscript, we will address the reviewer’s concerns with additional data and discussion as indicated above.*

## 3. Description of the revisions that have already been incorporated in the transferred manuscript

*N/A*

## 4. Description of analyses that authors prefer not to carry out

There are no analyses or discussion that we will not include. In one instance (as noted in section 2 in response to reviewer 2) we clarified that the 4sU data does not identify Oct4 enhancers due to the populations being investigated. The example data in reviewer figure noted in 2 and associated discussion hopefully addresses this point.
