# Supplementary material for: PRC2 promotes canalisation during endodermal differentiation
Source: PLoS Genet. 2025 Jan 30;21(1):e1011584. doi: 10.1371/journal.pgen.1011584 (PMC11813121; doi:10.1371/journal.pgen.1011584)
Supplement: Attachment — Submitted filename: Reponse to reviewers.docx [file pgen.1011584.s019.docx]

**Centre for Regenerative Medicine**

Institute for Regeneration and Repair

The University of Edinburgh

Edinburgh BioQuarter

5 Little France Drive

Edinburgh

EH16 4UU

**Tel**: +44 (0) 131 651 9500

**Fax**: +44 (0) 131 651 9501

regenerative-medicine@ed.ac.uk

**www.ed.ac.uk/regenerative-medicine**

**14^th^ January 2025**

**Re. Response to reviewers - PGENETICS-D-24-00986R1**

Dear reviewers and PLOS Genetics editorial team,

We thank the reviewers for their supportive and constructive feedback. We believe that we have addressed the final concern noted by reviewer 1 (and reiterated by the editor) as follows:

‘Please address the issue raised by Reviewer 1, that you "should not claim that gene activation does not require the loss of H3K27me3 at the gene promoter (TSS)." If this edit is made, we will be happy to accept the manuscript without further edits.’

To address this, we have updated the wording in the summary section and deleted the above statement entirely from the results section as requested. In addition, we have added raw data and computer code accession information to the manuscript and updated the funding information. These changes are highlighted in a tracked version of the manuscript and updated in the final submitted version of the manuscript.

On behalf of myself, Joshua Brickman and the other authors, I thank you for your consideration.

Yours sincerely,

Dr Rob Illingworth - Group Leader. Centre for Regenerative Medicine
